# Is Pakistan Well‑Positioned in the Global Health Security Scenario? An Exploratory Qualitative Study with Policy Experts and Public Health Professionals

**DOI:** 10.5334/aogh.4536

**Published:** 2024-11-21

**Authors:** Babar Tasneem Shaikh, Muhammad Ahmed Abdullah, Waleed Qaisar Shaikh, Nargis Yousuf Sattar, Shahzad Ali Khan

**Affiliations:** 1 Health Services Academy, Chak Shahzad, Park Road, Islamabad, Pakistan; 2 National University of Medical Sciences, Rawalpindi, Pakistan; 3 Central Institute of Family Medicine, Islamabad, Pakistan

**Keywords:** global health security, health system, health policy, Pakistan

## Abstract

*Background:* Pakistan’s health system is expected to take a proactive position in the global health security arena amidst its own indigenous structural and systemic challenges. Henceforth, understanding the intricacies of this system is vital for creating effective strategies to prevent, detect, and respond to health emergencies, ensuring regional stability and overall global health security.

*Objectives:* This study has ventured to understand the perspectives, hurdles, threats, and both international and national implications of Pakistan’s current health system capacity and the requisites for meeting global health security commitments.

*Methods:* This descriptive qualitative study, based on phenomenology theory, involved key informant interviews with 16 senior policy‑level experts and public health professionals from the public and private sectors, international non‑governmental organizations (NGOs), development partners, and United Nations (UN) bodies. Thematic analysis was employed to identify key themes related to Pakistan’s health system and its role in global health security.

*Findings:* The study uncovered significant insights into the strengths and weaknesses of Pakistan’s health system, the impact of the coronavirus disease 2019 (COVID‑19) pandemic, and challenges such as funding constraints and fragmented healthcare delivery. It also highlighted threats like antimicrobial resistance and emphasized the importance of international collaboration. Areas needing special attention include multi‑drug resistance, food safety in emergencies, surge capacity of the frontline workforce, patient safety to reduce healthcare‑associated infections, and strengthening points of entry.

*Conclusion:* The COVID‑19 pandemic has highlighted both the vulnerabilities and the potential within Pakistan’s health system. To enhance its contribution to global health security, Pakistan needs a national policy stance, targeted health system reforms, improved resource allocation, workforce development, and strengthened partnerships with development agencies.

## Key Messages

- Pakistan as one of the last reservoirs of polio and many other infectious diseases and is expected to play a vital role in global health security.
- COVID‑19 pandemic has unveiled both the vulnerabilities and the potential of Pakistan’s health system to deal with a crisis of such scale.
- Capacity building of health workforce and a concerted strategy for One Health with intersectoral collaboration is imperative.
- Health system should be geared up to improve the overall state of responsiveness and preparedness.

## Introduction

As borders become increasingly blurred, global health security (GHS) is growing in importance [1]. This shift underscores the need for strong public health systems that can prevent, detect, and respond to infectious disease threats, no matter where they originate [2]. Navigating the complex global burden of diseases, Pakistan, being the world’s fifth most populous country with a unique mixed health system, emerges as a significant player in global health security [3]. The pandemic has revealed the deep interconnectedness of our world, with the virus crossing countries and continents, illustrating the interdependence of national health [4]. In this context, Pakistan is not just addressing its own health challenges but is also part of a global community committed to protecting public health worldwide [6].

Pakistan continues to grapple with the challenge of polio, remaining one of the only two countries yet to fully eradicate this incurable disease [3]. The population also suffers from a range of infectious diseases, including tuberculosis, malaria, dengue, multidrug resistant (MDR) typhoid, and both acute and chronic hepatitis [9]. Additionally, Pakistan has the highest rate of diabetes in the world, with a high prevalence of related comorbidities such as hypertension, heart diseases, and dyslipidemias, compounding its public health challenges [7]. The coronavirus disease 2019 (COVID‑19) pandemic has underscored the impossibility of isolating ourselves from global health threats. The interconnected nature of our world necessitates a collective and coordinated effort to address health issues. For Pakistan, this means not only tackling domestic health problems but also recognizing its role as a reservoir of infectious diseases with global consequences [8]. Antimicrobial resistance (AMR) is another critical issue Pakistan must confront. Inadequate prescribing practices and specific farming methods contribute significantly to the global AMR crisis. The overuse and misuse of antibiotics in both human health and animal husbandry promote the development of drug‑resistant bacterial species, jeopardizing the efficacy of antibiotics and other antimicrobial medications [9, 10]. Enhancing prescribing practices and reducing antibiotic overuse in both agricultural and clinical settings are imperative to address this issue [11].

Being the member state of the World Health Organization (WHO) Eastern Mediterranean Region, Pakistan has had a Joint External Evaluation (JEE) in 2016 and then in 2023 to assess its core capacities and the functionality of its commitment to International Health Regulations (IHR) 2005, considering the One Health approach. Since then, the Government of Pakistan has taken on a stewardship role to guide its healthcare system towards being a more responsible global participant in health security [12, 13]. This requires strong governance, a solid national policy stance, effective rules and regulations, and their efficient implementation. To effectively address Pakistan’s health challenges and enhance global health security, a multifaceted strategy is essential. This strategy should comprise a strong support for immunization programs, an all‑inclusive workforce development plan, and sizeable investment in health systems research. Additionally, fostering international cooperation in health research and development is crucial. A strong and functional surveillance and monitoring systems for infectious diseases will not only improve the country’s ability to manage its health issues but also position Pakistan as a key contributor to global health security efforts [14]. By implementing these crucial steps, Pakistan can stand with the international community to embrace the mission of safeguarding global health.

This study aims to explore the understanding, perspectives, hurdles, threats, and both international and national implications of Pakistan’s current health system capacity and the requisites for meeting global health security commitments. Additionally, this research seeks to piece together the diverse elements that shape Pakistan’s role and define its position in the domain of global health security.

## Methodology

### Study design

This study employed a qualitative phenomenological approach to explore the understanding, perspectives, hurdles, threats, and implications of the state of affairs with regard to Pakistan’s current health system and the needs to meet for fulfilling global health security agenda. The phenomenological approach was chosen to gain deep insights into the lived experiences and perceptions of key stakeholders working for the health sector.

### Participants and sampling

We conducted 16 key informant interviews with various stakeholders across Pakistan. The respondents were purposely selected and comprised senior policy‑level experts and public health professionals in Islamabad, who have worked in GHS and One Health. This selection included public sector professionals, academicians, senior infectious disease physicians, and private sector and international NGO professionals. Nine interviews were conducted face‑to‑face in Islamabad, while the remaining seven were conducted via telephone with individuals working in different provinces. The sample size was determined on the basis of the principle of data saturation, ensuring that interviews were conducted until no new information emerged.

### Data collection

The interviews were conducted by all three investigators. Each interview lasted between 45 and 60 minutes. Interviews were primarily held at the participants’ offices or workplaces to facilitate a comfortable and convenient setting for the interviewees and to enable rapport building. All participants were explained the research objectives while obtaining informed consent. The interviews were audio‑recorded with the participants’ consent and transcribed verbatim within 24 hours to ensure accuracy and completeness of the data.

### Data analysis

We followed the Consolidated Criteria for Reporting Qualitative Research (COREQ) guidelines to ensure the rigor and transparency of the data collection and analysis process [15]. Thematic analysis was conducted using Braun and Clarke’s six‑step method [16]. The research team carefully read and re‑read the interview transcripts to fully familiarize themselves with the data. Initial codes were generated to label and categorize meaningful segments of data. Codes were then refined and grouped into broader themes that captured the essence of the data. These themes were reviewed to ensure they accurately represented the data and were coherent and distinct. The themes were defined and named to reflect the underlying concepts. Exemplars, or illustrative quotes, were identified to support each theme. The analysis was an iterative process, involving continuous refinement and validation of themes. Rigorous note‑taking was done during the interviews to capture detailed information. The research team engaged in regular discussions to validate the emerging themes and ensure the reliability and consistency of the analysis. These discussions helped to address any discrepancies and achieve a consensus on the interpretation of the data.

### Ethical considerations

Ethical approval for the study was obtained from the institutional review board at the Health Services Academy, Islamabad. Informed consent was obtained from all participants before conducting the interviews. Participants were assured of the confidentiality and anonymity of their responses, and they had the right to withdraw from the study at any time without any consequences.

By employing a phenomenological approach and following rigorous qualitative research standards, this study aims to provide a comprehensive understanding of the challenges and opportunities in Pakistan’s health systems and its role in global health security.

## Results

The participants provided comprehensive views that were categorized into several emergent themes. Table 1 presents the details of the participants.

**Table 1:** Basic demographic details of participants.

PARTICIPANT ID	AGE	GENDER	LOCATION	JOB ROLE	SECTOR	YEARS OF EXPERIENCE
P1	45	Male	Islamabad	Policy expert	Public sector	20
P2	38	Female	Karachi	Clinician	Private sector	15
P3	50	Male	Lahore	Pathologist	NGO	25
P4	42	Female	Peshawar	Public health professional	UN body	18
P5	36	Male	Quetta	Senior researcher	UN body	12
P6	48	Female	Islamabad	Policy expert	Public sector	22
P7	41	Male	Karachi	Clinician	Private sector	17
P8	44	Female	Lahore	Pathologist	NGO	19
P9	39	Male	Islamabad	Public health professional	Public sector	16
P10	37	Female	Islamabad	Senior researcher	Private sector	14
P11	53	Male	Islamabad	Policy expert	UN body	30
P12	47	Female	Karachi	Health system expert	WHO	21
P13	35	Male	Lahore	Pathologist	Public sector	10
P14	46	Female	Peshawar	Public health professional	NGO	20
P15	40	Male	Islamabad	Senior researcher	Private sector	15
P16	49	Female	Islamabad	Policy expert	Public sector	24

## Theme 1: Understanding of Global Health Security

Participants generally defined global health security as the ability to prevent, detect, and respond to infectious disease threats across borders. Many emphasized the interconnectedness of health systems worldwide; one particularly narrated that “a health threat in one country can quickly become a global issue.” (P11) There was a shared awareness of international frameworks, such as the International Health Regulations (IHR) and GHS, but opinions varied on Pakistan’s adherence to these standards.

## Theme 2: Strengths and Weaknesses of Pakistan’s Health Systems

The informants identified several strengths within Pakistan’s health systems, including robust immunization programs and strong community health initiatives. One participant highlighted, “Our polio eradication efforts, despite challenges, have built significant grassroots health infrastructure.” (P4) However, weaknesses were also prevalent, with frequent mentions of inadequate funding, poor infrastructure, and fragmented healthcare delivery. Another interviewee stated, “Our health system lacks cohesion and often fails to provide comprehensive care, especially in rural areas.” (P1)

## Theme 3: Impact of COVID‑19 on Health Systems

The COVID‑19 pandemic had a profound impact, serving as both a stress test and a catalyst for change. Participants shared that the pandemic exposed existing vulnerabilities but also led to rapid innovations, such as telemedicine and enhanced surveillance systems. “COVID‑19 forced us to rethink and innovate quickly,” one clinician remarked, “but it also highlighted the urgent need for a more resilient health infrastructure.” (P2)

## Theme 4: Challenges and Hurdles

Participants discussed numerous challenges hindering effective health system functioning, including limited funding, bureaucratic hurdles, and lack of trained healthcare professionals. One policy expert noted, “Funding is always a constraint, but the inefficiencies in how resources are allocated compound the problem.” (P6) Additionally, the disparity between urban and rural healthcare was a recurring theme, with rural areas often facing severe shortages of medical facilities and personnel.

## Theme 5: Threats to Health Security

Emerging health threats such as drug‑resistant tuberculosis and recurrent outbreaks of diseases such as dengue and typhoid were major concerns. One public health professional warned, “AMR is a ticking time bomb for our healthcare system.” (P9) The participants emphasized the need for urgent action to address these threats through better regulation, improved prescription practices, and public awareness campaigns.

## Theme 6: Role of International Collaboration

International collaboration was seen as vital to strengthening Pakistan’s health systems. Participants appreciated existing partnerships with organizations like the WHO and various UN bodies, but they also called for deeper and more strategic alliances. “International support has been crucial,” said one senior researcher, “but we need more tailored collaborations that address our specific challenges.” (P10)

## Theme 7: Policy and Regulation

Current policies are often criticized for being outdated or poorly implemented. Many participants suggested that policy reforms are essential to improving health security. “Our regulatory framework needs an overhaul,” stated one clinician, “to better control the use of antibiotics and manage public health threats effectively.” (P7)

## Theme 8: Antimicrobial Resistance (AMR)

AMR was a prominent theme, with many participants highlighting it as a significant and growing threat. Poor prescription practices and unregulated use of antibiotics in agriculture were frequently mentioned issues. A pathologist shared, “We are already seeing cases where infections are resistant to multiple drugs, and this trend is worrying.” (P8)

## Theme 9: Resource Allocation and Management

Resource allocation and management emerged as critical areas needing improvement. Participants expressed concerns about the inefficient use of available resources and called for better management practices. “It’s not just about how much we have, but how well we use it,” emphasized one policy expert, suggesting that strategic investments and accountability could enhance system performance. (P16)

## Theme 10: Future Directions and Recommendations

Finally, participants offered various recommendations for the future. They called for strengthened health policies, improved resource allocation, enhanced international collaboration, and a comprehensive strategy for workforce development, especially the frontline workers. “We have the potential to turn things around,” one senior infectious disease physician remarked, “but it requires a concerted effort from all stakeholders to build a resilient and effective health system.” (P13)

## Discussion

The COVID‑19 pandemic underscored the intricate and interconnected challenges posed by globalization, significantly affecting health equity, economic security, and environmental justice, particularly for marginalized communities and people of color in the United States [17]. These disparities became evident through limited access to quality healthcare, affordable housing, and financial resources, with documented and undocumented immigrants facing compounded difficulties. Therefore, while our study emphasized the importance of aligning health security strategies with global efforts, it also highlighted the urgent need to address the underlying socio‑economic factors that perpetuate inequities within domestic healthcare systems. The analysis revealed notable strengths within Pakistan’s health system, particularly in immunization programs and the deployment of community health workers, providing a solid foundation for further improvements. However, significant weaknesses, such as inadequate funding, poor infrastructure, and fragmented healthcare delivery, pose substantial barriers to achieving optimal health outcomes. Critical areas needing attention include antimicrobial resistance, particularly multidrug resistance, food safety during emergencies, surge capacity of the frontline workforce, patient safety to reduce healthcare‑associated infections, and strengthening points of entry [13]. Addressing these weaknesses will require targeted investments and policy reforms to create a more cohesive and resilient health system [18].

The COVID‑19 pandemic initially exposed Pakistan’s lack of standardized operating procedures, leading to significant challenges in response efforts. The government had to import testing kits, and violations of lockdown measures and standard operating procedures (SOPs) contributed to a rapid surge in cases, overwhelming the healthcare system. Ethical concerns arose regarding discussions on achieving “herd immunity” and the potential sacrifice of immune‑compromised individuals for population‑wide immunity. Additionally, limited testing capacity hindered effective containment efforts, raising doubts about the government’s ability to manage the healthcare crisis. Our findings align with other studies highlighting these challenges, emphasizing the urgent need for targeted investments and policy reforms to strengthen Pakistan’s healthcare system and enhance its capacity to combat pandemics efficiently [19]. The COVID‑19 pandemic has had a profound impact on Pakistan’s health systems, serving as both a stress test and a catalyst for innovation. The rapid adoption of telemedicine and enhanced surveillance systems during the pandemic demonstrates the potential for agile responses to health crises. However, the pandemic also exposed critical vulnerabilities, particularly in resource allocation and crisis management. These insights underscore the need for sustained efforts to bolster health infrastructure and emergency preparedness [20].

Limited funding, bureaucratic hurdles, and a shortage of trained healthcare professionals are the challenges which are exacerbated by the significant disparity between urban and rural healthcare services [21]. Similar issues were found in another study, where thematic analysis showed that Pakistan’s hospitals’ lack specialized human resource (HR) departments manned by qualified HR professionals and that it had a detrimental impact on HR management functions and practices and increased complexity and confusion. Limited managerial autonomy over important HR matters and centralized decision‑making at the provincial level made this worse. Though the Pakistani public healthcare sector has several obstacles, a better HR management might have a big impact on changing staff attitudes and actions so that more patients receive high‑quality care [22]. To address these issues, a comprehensive approach is necessary, encompassing policy reforms, increased funding, and strategic investments in healthcare workforce development and infrastructure improvements. Urgent attention is needed to address the rise of AMR, fueled by poor prescription practices and unregulated antibiotic use in agriculture. However, implementing the National Action Plan (NAP) for AMR faces challenges such as limited diagnostic facilities, financial resources, and the continued excessive use of antimicrobials in hospitals and their unrestricted sale in communities [23]. Moreover, livestock farming in Pakistan is consuming alarmingly high amount of antimicrobials critical for human health. These data provide a reference for closer surveillance and empirical research aiming to reduce antibiotic use in food animals [24]. Healthcare authorities must revise and prioritize NAP objectives through robust policies, antimicrobial stewardship interventions, and infection prevention and control practices, requiring comprehensive strengthening of the healthcare system.

International collaboration is essential for strengthening Pakistan’s health systems. Current partnerships with WHO and other development organizations should evolve into deeper, more strategic alliances. By fostering more synchronized collaborations that address specific challenges unique to Pakistan, the country can enhance its ability to respond to health threats and integrate more effectively into the global health security framework [25]. Moreover, the need for policy reforms was a recurrent theme, with recommendations focusing on updating regulatory frameworks to better control antibiotic use and manage public health threats effectively. Ensuring that these policies are not only well‑designed but also rigorously implemented will be crucial for improving health security [3, 26]. Resource allocation and management as areas requiring significant improvement within Pakistan’s healthcare system. Concerns were raised regarding the inefficient utilization of available resources, emphasizing the need for better management practices. To optimize the system’s performance, strategic investments, efficient resource utilization, and accountability mechanisms are deemed necessary [18]. Moreover, the study underscores the central focus of the current regime on public healthcare. It is evident that healthcare spending plays a vital role in shaping health outcomes in Pakistan, contributing significantly to increased life expectancy and reduced mortality rates. Given these findings, the government is urged to allocate sufficient resources toward the acquisition of the latest healthcare technologies and equipment, thereby enhancing health outcomes nationwide [27].

## Conclusion

Pakistan has the potential to transform its health system by addressing these critical areas, thereby enhancing its preparedness and readiness for future health emergencies of this scale. It is crucial to recognize the importance of equitable partnerships and bidirectional learning while engaging with the global development community. By embracing these principles, Pakistan can shift from being seen as a potential threat to becoming a proactive partner in safeguarding global health and well‑being. The COVID‑19 pandemic has highlighted both the vulnerabilities and the innovative potential within these systems. To move forward, it is essential to address challenges such as antimicrobial resistance, improve resource management, and foster international collaboration. These goals would be achievable if Pakistan focused on building a national health security framework with standardized crisis response protocols, sustained investment in healthcare infrastructure, and well‑targeted programs for containing antimicrobial resistance, the weakest core competency identified in JEE 2023. Supportive policies include the creation of incentives to promote openness in sharing data with its global partners, building the capacity of health professionals working in rural and underserved areas, and routine worker training to increase the resiliency of the health care sector. By implementing targeted policy reforms and enhancing public health education, Pakistan can strengthen its health systems and contribute more effectively to global health security. This research emphasizes the need for a cohesive, well‑funded, and resilient health infrastructure to tackle both national and international health challenges.

## Data Availability

The datasets used and/or analyzed during the current study are available from the corresponding author on reasonable request.
